# An EMD-Based Adaptive Client Selection Algorithm for Federated Learning in Heterogeneous Data Scenarios

**DOI:** 10.3389/fpls.2022.908814

**Published:** 2022-06-09

**Authors:** Aiguo Chen, Yang Fu, Zexin Sha, Guoming Lu

**Affiliations:** School of Computer Science and Engineering, University of Electronic Science and Technology of China, Chengdu, China

**Keywords:** distributed conjoint analysis, federated learning, adaptive client selection, statistical heterogeneity, machine learning

## Abstract

Federated learning is a distributed machine learning framework that enables distributed nodes with computation and storage capabilities to train a global model while keeping distributed-stored data locally. This process can promote the efficiency of modeling while preserving data privacy. Therefore, federated learning can be widely applied in distributed conjoint analysis scenarios, such as smart plant protection systems, in which widely networked IoT devices are used to monitor the critical data of plant production to improve crop production. However, the data collected by different IoT devices can be dependent and identically distributed (non-IID), causing the challenge of statistical heterogeneity. Studies have also shown that statistical heterogeneity can lead to a marked decline in the efficiency of federated learning, making it challenging to apply in practice. To promote the efficiency of federated learning in statistical heterogeneity scenarios, an adaptive client selection algorithm for federated learning in statistical heterogeneous scenarios called ACSFed is proposed in this paper. ACSFed can dynamically calculate the possibility of clients being selected to train the model for each communication round based on their local statistical heterogeneity and previous training performance instead of randomly selected clients, and clients with heavier statistical heterogeneity or bad training performance would be more likely selected to participate in the later training. This client selection strategy can enable the federated model to learn the global statistical knowledge faster and thereby promote the convergence of the federated model. Multiple experiments on public benchmark datasets demonstrate these improvements in the efficiency of the models in heterogeneous settings.

## Introduction

With the rapid development of computation and storage capabilities, IoT devices are now widely applied in multiple areas, such as health monitors, smart homes, and smart plant protection systems. These devices are used to collect critical data during operation and provide a statistical foundation for services, such as health prediction, house management, or the direction of plant production. However, the broad deployment of these devices will lead to the expansion of the data scale and the general growth in the demand for privacy preservation of sensitive data, and the traditional centralized machine/deep learning mode that collects all data in a central node to train a statistical model has difficulty meeting demand. Specifically, transmitting distributed-stored data to a central node and training a model on that scale of data can lead to long computation and communication times. In addition, privacy leakage of sensitive data may occur during transmission. Therefore, federated learning (Konečný et al., 2015; McMahan et al., 2017a,b), a distributed machine learning framework that involves a central server and multiple distributed nodes, is proposed in this study to address the challenges that face centralized frameworks. Federated learning enables distributed nodes with computation and storage capabilities, such as mobile phones, IoT devices, and laptops, to train statistical models using local data for each communication round. Only the model parameters are transmitted to a central server. The central server then aggregates these local models to generate the new federated model for the next communication round. Federated learning can provide more efficient construction of the global model and privacy of data because it decentralizes the computation among distributed clients and keeps the distributed-stored data locally.

However, in practical scenarios, the differences in device type, geographical location of deployment, and other factors will make the collected data non-IID, resulting in the challenge of statistical heterogeneity. Statistical heterogeneity is known to negatively impact multiple aspects of federated learning compared with IID data, like performance, efficiency, data privacy, etc. Non-IID data can have various features or label distributions, which causes marked differences between local models and thus leads to unstable convergence and low efficiency during federated model training. To address the influence of statistical heterogeneity on the efficiency of federated learning, many studies have investigated three aspects of this topic: restricting model divergence, decreasing communication cost, and customized client selection methods. Regarding restricting model divergence, some studies have focused on improving data quality, such as data sharing (Zhao et al., 2018) and data enhancement (Jeong et al., 2018).

On the contrary, other studies have investigated model training by modifying the model aggregation method (McMahan et al., 2017a), customizing the loss function (Li et al., 2020), and combining enhancement methods, including reinforcement learning (Pang et al., 2020; Wang et al., 2020) and knowledge distillation (Li and Wang, 2019), with federated learning. The relation between statistical heterogeneity and the Earth's mover distance (EMD) is presented in Zhao et al. (2018). The EMD is essentially used to measure the distance between two distributions. Experimental results indicate that EMD could be an ideal index of statistical heterogeneity by calculating the EMD between the local data distribution on a client and the global data distribution. There is also some constructive work regarding decreasing communication costs, such as model compression (Sattler et al., 2019) and dynamic computing of the rounds of local updates (Wang et al., 2019). Regarding the customized client selection method, recent studies have proposed creatively customized methods (Nishio and Yonetani, 2019; Wang et al., 2020; Shen et al., 2022; Zhang et al., 2022; Zhao et al., 2022) to accelerate the convergence of federated learning and thereby promote efficiency. Moreover, Xiong et al. (2021) carried out an innovative exploration of privacy protection in FL with non-IID data and proposed a differential-privacy-based algorithm called 2DP-FL, which has advantages in privacy protection, learning convergence, and model accuracy.

In this study, we propose an adaptive client selection algorithm for federated learning called ACSFed based on the finding of correlations between EMD and statistical heterogeneity in Zhao et al. (2018). In ACSFed, the index measuring the data and training quality of each client, called cumulative model strength, is calculated based on each client's EMD and previous training performance. Then, a probability matrix representing the probability of each client being selected in each communication round is maintained and dynamically updated based on the cumulative model strength of each client. ACSFed can fully consider the data and previous training quality of each client to update the probability matrix so that clients with strong statistical heterogeneity or poor training performance will be more likely to be selected for training, thereby enabling the federated model to learn the unknown information or knowledge with poor learning performance much faster. After the federated model has learned sufficient knowledge of the global data, the convergence of the federated model speeds up markedly, and multiple experiments have shown that ACSFed can converge faster than FedAvg. The primary contributions of this paper can be summarized as follows:

1. An index measuring the statistical heterogeneity and previous training performance of a client called cumulative model strength is first proposed.
2. An adaptive client selection algorithm called ACSFed for federated learning based on cumulative model strength is proposed in this study. The probability of a client being selected to train the statistical model is dynamically updated.
3. Through ACSFed, the performance of federated learning in statistical heterogeneity scenarios can be promoted compared with current methods.

The remainder of this paper is organized as follows. Section Related Work describes the background of federated learning research in statistical heterogeneity scenarios. Section Problem Definition describes the problem that current methods face in statistical heterogeneity scenarios. Then, Section Method describes the structure and principles of the proposed approach. The theoretical analysis and experimental results are both shown in Section Evaluation. Finally, the conclusions of this study are provided in Section Conclusion.

## Related Work

Federated learning was first introduced by McMahan et al. (2017b). Its baseline algorithm is the federated stochastic gradient descent (FedSGD), enabling each client to execute a round of SGD locally and upload the model to a central server for aggregation. Although FedSGD can solve the challenges of privacy leakage of sensitive data and achieve the same accuracy of centralized model training when the data are IID, frequent model uploading and distribution have markedly increased the communication burden, resulting in the problem of efficiency.

In 2017, an improved algorithm called FedAvg (McMahan et al., 2017a) was proposed and allowed clients to synchronously execute several rounds of SGD before uploading the model to a central server for model aggregation, which efficiently decreases the communication rounds and thus promotes the efficiency of federated learning. The convergence of FedAvg is theoretically proven in Li et al. (2019), and experiments on public benchmark datasets also demonstrate that FedAvg has ideal convergence and robustness. However, Zhao et al. (2018) found that the performance and efficiency of FedAvg markedly shrink as statistical heterogeneity increases. Moreover, the relation between the EMD of statistical heterogeneity has been identified and implies that EMD can act as an ideal index of statistical heterogeneity. Further, Zhang et al. (2020) has used EMD to measure the degree of statistical heterogeneity and proposed a personalized federated learning model training algorithm, which outperforms existing alternatives. The results of Zhao et al. (2018) and Zhang et al. (2020) have proved that EMD can effectively measure statistical heterogeneity and can be applied to promote the performance and efficiency of federated learning.

Focusing on promoting the efficiency of federated learning, Wang et al. (2019) introduced adaptive federated learning that can dynamically compute the rounds of local updates for each client in resource-constrained edge computing systems. Compared with methods where the communication step is fixed, faster convergence can be achieved. Similarly, Huang et al. (2020) proposed an adaptive enhancement method called LoAdaBoost, which optimizes the first half of the local update rounds, and only those clients with low performance would continue training. This strategy can markedly reduce the expectation of local training rounds for each client, thereby promoting the efficiency of federated learning. In addition, considering communication costs, Konečný et al. (2016) markedly reduces communication costs via model compression, decreasing the uploaded model's size.

Similarly, Sattler et al. (2019) proposed a compression framework called sparse ternary compression (STC), which extends the existing compression technique by enabling downstream compression and internalization and optimal Golomb encoding of the weight updates. Moreover, Cai and Zheng (2018) considers energy conservation and privacy preservation and proposes an energy-efficient mechanism for data transmission. Besides energy conservation and privacy preservation (Cai and He, 2019; Cai et al., 2019), also consider data utility, and, respectively, proposed two mechanisms that can further preserve the results while protecting privacy. Additionally, Asad et al. (2020) introduced an algorithm combined with model compression and parameter encryption, effectively reducing communication overhead while protecting model security. Besides directly reducing communication costs, the efficiency of federated learning could also be improved by resource optimization. For example, Nishio et al. (2013), Sardellitti et al. (2015), and Yu et al. (2016) minimized the computation time and resource consumption based on the joint optimization of heterogeneous data, computation, and communication resources. In contrast, Nishio and Yonetani (2019) maximized the efficiency of federated model training through client selection based on resources, network conditions, and computation capability, and experiments have proven efficiency enhancement. However, these methods might markedly increase the computational burden in the distributed nodes, depending on the environment settings of federated learning, making the applicability of these methods not ideal.

In addition to improving the efficiency of federated learning by reducing communication cost and resource use, there are also methods that design client selection strategies to promote efficiency. For example, Chen et al. (2020) proposed an approximation algorithm that effectively improves efficiency and reduces the communication complexity by selecting some clients and allowing them to upload their updates in each round of training. Still, this method lacks the consideration of statistical heterogeneity. In 2022, Shen et al. (2022) proposed a novel stratified client selection scheme that develops stratification based on clients' local data distribution to derive approximate homogeneous strata for better selection in each stratum and can reduce the variance of the selected subset of clients for the pursuit of better convergence and higher accuracy. Experimental results show that their approach allows for better performance than existing methods and is compatible with prevalent federated learning algorithms. Similarly, Zhao et al. (2022) proposed FedNorm, a client selection framework that finds the clients that provide essential information in each round of model training and reduces energy cost by decreasing the number of participating clients. Evaluation results demonstrate that those algorithms outperform existing federated learning client selection methods in various statistical heterogeneity scenarios. In addition, there is also the idea of combining reinforcement learning with client selection. For example, Wang et al. (2020) proposed Favor. This experience-driven control framework intelligently chooses the client devices to participate in each round of federated learning to counterbalance the bias introduced by non-IID data and speed up convergence. However, that method requires specific experience to obtain ideal performance. Inspired by Multi-Agent Reinforcement Learning (MARL) in solving complex control problems, Zhang et al. (2022) presented FedMarl, a MARL-based federated learning framework that performs efficient run-time client selection. Experiments have shown that FedMarl can promote model accuracy with much lower processing latency and communication costs. However, FedMarl might increase the computational burden in the nodes, making it unsuitable for computation-ability-constrained scenarios. Additionally, Cho et al. (2020) found that selecting clients with higher local loss can improve convergence speed, and they proposed a client selection algorithm called Power-of-Choice based on their discovery. Compared with the random client selection algorithm, the convergence speed of the Power-of-Choice algorithm is significantly improved. Similarly, Zhang et al. (2021) utilizes weight divergence to identify the non-IID degree of clients, and proposes an efficient federated learning algorithm called CSFedAvg, which speeds up the training by selecting clients with less non-IID data to train the model more frequently. Although the speed of convergence and training accuracy is improved by applying CSFedAvg, it does not guarantee to cover all clients, resulting in the lack of completeness of the global data used for training.

Thus, although current studies have investigated improving the performance of federated learning in statistical heterogeneity scenarios, problems of higher computing and communication burdens and difficulties in practical application exist in recent research. Therefore, an improved method that can suppress or solve the above issues while retaining or improving performance must be studied.

## Problem Definition

This paper proposes an adaptive client selection algorithm for federated learning in statistical heterogeneity scenarios. Federated learning is a distributed machine learning framework in which a set *C* = {1, 2, ..., *K*} of *K* distributed clients owns a set of local data sampled from the global dataset *D* with M categories in total. The resulting statistical model is asynchronously trained with the same structure using local data. The optimization of federated learning is to train a suitable global parameter vector ω, which can minimize the total loss of the distributed clients and can be quantified as follows:


(1) $$\begin{array}{r} \underset{\omega }{\text{minimize}}F_{f}\left(\omega \right)=\sum_{i=1}^{K}\frac{\left|D_{i}\right|}{\left|D\right|}f_{i}\left(\omega ,D_{i}\right) \end{array}$$

In IID scenarios, the local optimization object of any client is an unbiased estimate of the centralized optimization object *F*_*c*_(ω) because the data distribution of these clients is the same as the global data distribution *P*. Therefore, instead of enabling all clients to participate in training, only a portion of clients can be selected randomly with a specific fraction to execute local updates in the model training of federated learning. This strategy can promote the efficiency of federated learning because it can markedly decrease the load and number of network transmissions.

However, *f*_*i*_(ω, *D*_*i*_) could be an arbitrary approximation to *F*_*c*_(ω) when data across the clients is non-IID, which *P*_*i*_ can be markedly different *P*, leading to the deviation between the federated model and centralized-trained model and a marked decrease in the performance of the federated model. The convergence of the global model can be unstable due to the non-IID data because it is difficult for the model to learn the knowledge of the global data distribution by randomly selecting clients to participate in training. Therefore, we use ACSFed to solve the problem mentioned above. The goal of ACSFed is to make clients with heavy statistical heterogeneity and poor model performance selected with a larger possibility in the early training rounds, thereby enabling the global model to learn unknown data information as quickly as possible and providing more stable, faster convergence than random client selection. The theory and design of ACSFed are described in the next section.

## Method

### Definition of Cumulative Model Strength

In this section, the structure and principles of ACSFed will be shown in detail. In ACSFed, a probability matrix representing the probability of each client being selected is maintained and dynamically updated after each communication round. The updated probability is based on an index called the cumulative model strength of each client, which was first proposed by this study and considered the statistical heterogeneity and previous training performance. The calculation of cumulative model strength is defined as follows:


(2) $$\begin{array}{r} h_{t}^{\left(i\right)}\leftarrow \rho \cdot h_{t-1}^{\left(i\right)}+\frac{1-\rho }{EM{D_{i}}^{*}||Loss_{i}|{|}_{2}^{2}},i=1,2,....,N \end{array}$$

where $h_{t}^{\left(i\right)}$ and $h_{t-1}^{\left(i\right)}$ represent the cumulative model strength of the client *i* at the current and the previous communication rounds, respectively; *Loss*_*i*_ denotes the training loss; ρ represents the attenuation coefficient; *EMD*_*i*_ is the index of statistical heterogeneity on the client *i*, and *N* is the total number of clients. Therefore, a client's latest cumulative model strength depends on its history cumulative model strength and current training status. This calculation method uses an exponential-weighted average. It is widely applied in the optimization algorithms of deep learning, such as RSMProp (Tieleman and Hinton, 2012), which considers the history and current gradient information to adjust the learning rate of deep learning dynamically. In the exponential-weighted average, the attenuation coefficient ρ controls the acceptance ratio of history information. *EMD*_*i*_ is used to measure the degree of statistical heterogeneity in the client *i* by calculating the distance between the local data distribution *q*_*i*_ and the global data distribution *p*. Specifically, it is calculated as follows:


(3) $$\begin{array}{r} EMD_{i}\left(p,q_{i}\right)=\underset{\gamma \tilde{\;}\prod \left(p,q_{i}\right)}{\inf}{\text{E}}_{\left(x,y\right)\tilde{\;}\gamma }\left[||x-y|{|}_{2}\right] \end{array}$$

where ∏(*p, q*_*i*_) is the set of all possible joint distributions that combine the distributions *p* and *q*_*i*_. Then, for a specific joint distribution γ belonging to ∏(*p, q*_*i*_), a sample pair *x* and *y* can be sampled, and their distance can be calculated, thereby calculating ${\text{E}}_{\left(x,y\right)\tilde{\;}\gamma }\left[||x-y|{|}_{2}\right]$, which is the expectation of the distance between sample pairs under the joint distribution γ. Finally, the minimum expected value of the sample pair over all possible joint distributions is determined to be the EMD between distributions *p* and *q*_*i*_.

At the beginning of training, the cumulative model strength of each client is 0, and the values in the probability matrix are initialized to $\frac{1}{N}$, indicating randomly selecting clients. After every communication round, clients participating in the training update the values of cumulative model strength and then send them to the central server.

### Adaptive Probability Updating Method

In the central server, a set *H*_*t*_ is maintained, which contains the latest cumulative model strength of each client and is defined as follows:


(4) $$\begin{array}{r} H_{t}=\left\{h_{t}^{\left(1\right)},h_{t}^{\left(2\right)},...,h_{t}^{\left(N\right)}\right\} \end{array}$$

Only the cumulative model strength values of clients participating in the training are updated, while the rest remain unchanged. Then, the central server updates the probability matrix based on *H*_*t*_ which is:


(5) $$\begin{array}{r} matrix\text{\_}prob=matrix\text{\_}porb+\frac{EMD^{*}||Loss|{|}_{2}^{2}}{\sqrt{H_{t}+\varepsilon }} \end{array}$$

where ε is applied to avoid the situation in which the denominator is 0. As shown in Formula (10), the client with a larger cumulative model strength will have a smaller probability of being selected again because the model's performance on the client is sufficient. The probability of a client being selected can markedly increase when its cumulative model strength is 0, indicating that it has not participated in previous training and should be more likely to be chosen in the following training epoch. Finally, normalization is applied to ensure that the sum of all possible values in the matrix is equal to 1 and is calculated as:


(6) $$\begin{array}{r} matrix\text{\_}prob=\frac{matrix\text{\_}porb}{\sum_{i=1}^{N}matrix\text{\_}porb_{i}} \end{array}$$

where *matrix*_*porb*_*i*_ is the client's probability *i*, and then the clients participating in the following training epoch are selected based on the normalized probability matrix. Based on the theory and design demonstrated above, the process of ACSF is shown in Algorithm 1.

**Algorithm 1:** Adaptive client selection enabling federated learning (ACSFed). *K* clients are selected from *N* clients with fraction *c*; η is the learning rate, *P* is the probability matrix; *H* is the cumulative model strength matrix.

**Central Server:**
Initialize model ω_0_, *P* and *H*
*K*←max(*c*·*N*, 1)
**while** t in total communication rounds do:
if t=0:
client_set = {randomly selected *K* clients}
else:
client_set = {select *K* clients based on probability matrix *P*}
**for** each client *i* in client_set parallel **do:**
Transmit ω_*t*_ to client *i*
Receive $\omega_{t+1}^{i},\;||loss_{i}|{|}_{2}^{2}$, *emd*_*i*_ from client *i*
**end for**
$H_{i}\leftarrow f\left(||loss_{i}|{|}_{2}^{2},emd_{i}\right)$
*P*←*g*(*P, H*)
**end while**
**Distributed Client:**
Receive model ω_*t*_ from the central server
Calculate *emd*_*i*_ based on local data distribution
Initialize loss list *loss*_*i*_ = {}
Θ← {split local data into batches with size *B*}
**for** local epoch 1,2,…, E **do:**
**for θ∈Θ do:**
Add training loss to*loss*
ω_*t*_←ω_*t*_-η∇*g*(ω_*t*_, θ)
**end for**
**end for**
$\omega_{t+1}^{i}\leftarrow \omega_{t}$
Calculate$||loss_{i}|{|}_{2}^{2}$
Transmit $\omega_{t+1}^{i}||loss_{i}|{|}_{2}^{2}$ and *emd*_*i*_ to Central Server

After introducing the theory and design of the ACSFed algorithm, the theoretical analysis of ACSFed regarding the convergence of federated learning in statistical heterogeneity scenarios is performed and is described in the next section.

### Analysis of the Convergence of ACSFed

In this section, the theoretical analysis of FedAvg and ACSFed in statistical heterogeneity scenarios is performed. In statistical heterogeneity scenarios, the data across the distributed clients is non-IID. Assuming that the data distribution of the client *i* is *P*_*i*_ when the data distribution across the clients is IID (*P*_*i*_ = *P*), the expectation of the local optimization object in any client is an unbiased estimate of the centralized optimization object *F*_*c*_(ω):


(7) $$\begin{array}{l} \begin{array}{ccc} E_{D_{i}\tilde{\;}P_{i}}[f_{i}(\omega ,D_{i})] & = & F_{c}(\omega ) \end{array}\\ \begin{array}{cc} \;= & \frac{1}{\left|D\right|}{\sum^{\text{​}}}_{m=1}^{M}P(y=m)\cdot E_{x|y=m}[l(\omega ,x)] \end{array} \end{array}$$

Then, we calculate the expectation of the global federated learning optimization object *F*_*f*_(ω) as follows:


(8) $$\begin{array}{l} E[F_{f}(\omega )]=E[{\sum^{\text{​}}}_{i=1}^{K}\frac{\left|D_{i}\right|}{\left|D\right|}\cdot \frac{1}{\left|D_{i}\right|}l(\omega ,D_{i})]\\ =\frac{1}{\left|D\right|}E[{\sum^{\text{​}}}_{i=1}^{K}l(\omega ,D_{i})]=\frac{1}{\left|D\right|}l(\omega ,D)\\ =F_{c}(\omega ) \end{array}$$

Equation (8) shows that the expectation of federated learning optimizing object *F*_*f*_(ω) is equal to centralized optimization object *F*_*c*_(ω) when data across the clients is IID. Therefore, instead of enabling all clients to participate in training, only a portion of clients can be selected randomly with a specific fraction to execute local updates in the model training of federated learning because the local optimization object of any client is an unbiased estimate of *F*_*c*_(ω). However, *f*_*i*_(ω, *D*_*i*_) could be an arbitrary approximation to *F*_*c*_(ω) when *P*_*i*_ is different from *P*, leading to the deviation between the federated model and centralized-trained mode, and *E*[*F*_*f*_(ω)] is no longer equal to *F*_*c*_(ω). The convergence of the global model can be unstable due to the non-IID data because it is difficult for the model to learn the knowledge of the global data distribution by randomly selecting clients to participate in training. Therefore, statistical heterogeneity must be considered when selecting clients to participate in model training every communication round. The previous training performance should also be regarded to make the model learn the global knowledge faster. Specifically, the client with heavier statistical heterogeneity and poor training performance should be more likely to be selected to accelerate the convergence federated model, and this client choosing strategy is based on the research of Cho et al. (2020), which gives the theory of convergence of federated learning:

***Theorem 5.1:*** The expectation of the difference between the training parameter and the convergence parameter is shown below, and the smaller it is, the faster convergence will be achieved:


(9) $$\begin{array}{l} E(F_{f}(\omega_{t})-F_{f}^{*})\leq \frac{1}{T+\gamma }[\frac{4L(32\tau^{2}G^{2}+\sigma^{2}/m)}{3\mu^{2}\bar{\rho }}\\ +\frac{8L^{2}\Gamma }{\mu^{2}}+\frac{L\gamma ||\omega_{0}-\omega^{*}|{|}^{2}}{2}]+\xi \;\cdot \;(\frac{\tilde{\rho }}{\bar{\rho }}-1) \end{array}$$

where Γ is the difference between centralized optimization object and federated optimization object; γ, μ are parameters that control the learning rate; and the critical factor is $\overset{\text{\_}}{\rho }$, which is called Selection Skew, and it is defined as follows:


(10) $$\bar{\rho }\underline{\underline{\Delta }}\underset{\omega_{t}}{\min}\frac{E_{S(\pi )}\left[\frac{1}{m}\sum_{k\in S(\pi )}\left(f_{k}(\omega_{t},D_{k})-f_{k}^{*}\right)\right]}{F_{f}(\omega_{t})-\sum_{k=1}^{K}p_{k}f_{k}^{*}}$$

where *S*(π) is the client selection strategy and assumes the strategy would select *m* clients to participate in the training; $f_{k}^{*}$ is the final local optimizing object representing the training has converged and ω_*t*_ is the model parameter to be evaluated during training.

Then as can be concluded in ***Theorem 5.1***, the larger $\overset{\text{\_}}{\rho }$ is, the smaller the expectation of the difference between the training parameter and convergence parameter, indicating that faster convergence will be achieved. The client selection strategy of ACFed would more likely choose clients with heavier statistical heterogeneity, worse training performance, or untrained operation; therefore, the *f*_*k*_(ω_*t*_, *D*_*k*_) of each client will be much bigger than simply random selection, and thereby the $\overset{\text{\_}}{\rho }$ will be bigger. Since the strategy of ACSFed will make $\overset{\text{\_}}{\rho }$ bigger than the strategy that randomly selects clients, according to ***Theorem 5.1***, faster convergence will be obtained by ACSFed. After the theoretical analysis of ACSFed, performance experiments will be introduced in Section Evaluation.

## Evaluation

In this section, the results of the experiments are reported. Three public benchmark datasets are used for evaluation: MNIST, Fashion MNIST, and CIFAR 10, and the details of these datasets are shown in Table 1.

**Table 1:** Details of the dataset.

**Dataset**	**Category**	**Size**	**Description**
		**(length, width, channel)**	
MNIST	10	28 × 28 × 1	Hand-writing number image
Fashion MNIST	10	28 × 28 × 1	Wearing image
CIFAR 10	10	32 × 32 × 3	Common things image

As shown in Table 1, the three datasets are all images, and MNIST and Fashion MNIST are grayscale images (with only one channel), while CIFAR 10 is a color image (with three channels). In terms of the models used to learn features and classification, a seven-layer convolutional neural network (CNN) is used in MNIST and Fashion MNIST, while a nine-layer CNN is used in CIFAR 10. For the baseline algorithm, FedAvg, which selects clients randomly in each communication round, is chosen for the performance comparison. Two statistical heterogeneity scenarios are set in the experiment, including a 2-class non-IID scenario (each client has two categories of images) and a 1-class non-IID scenario (each client has only one category of images). To determine the best attenuation coefficient ρ, several experiments are performed using different ρ. For the evaluation, two factors are selected (accuracy reduction and convergence time), and the results are shown in the following experimental figures and table.

**Figure 1:**
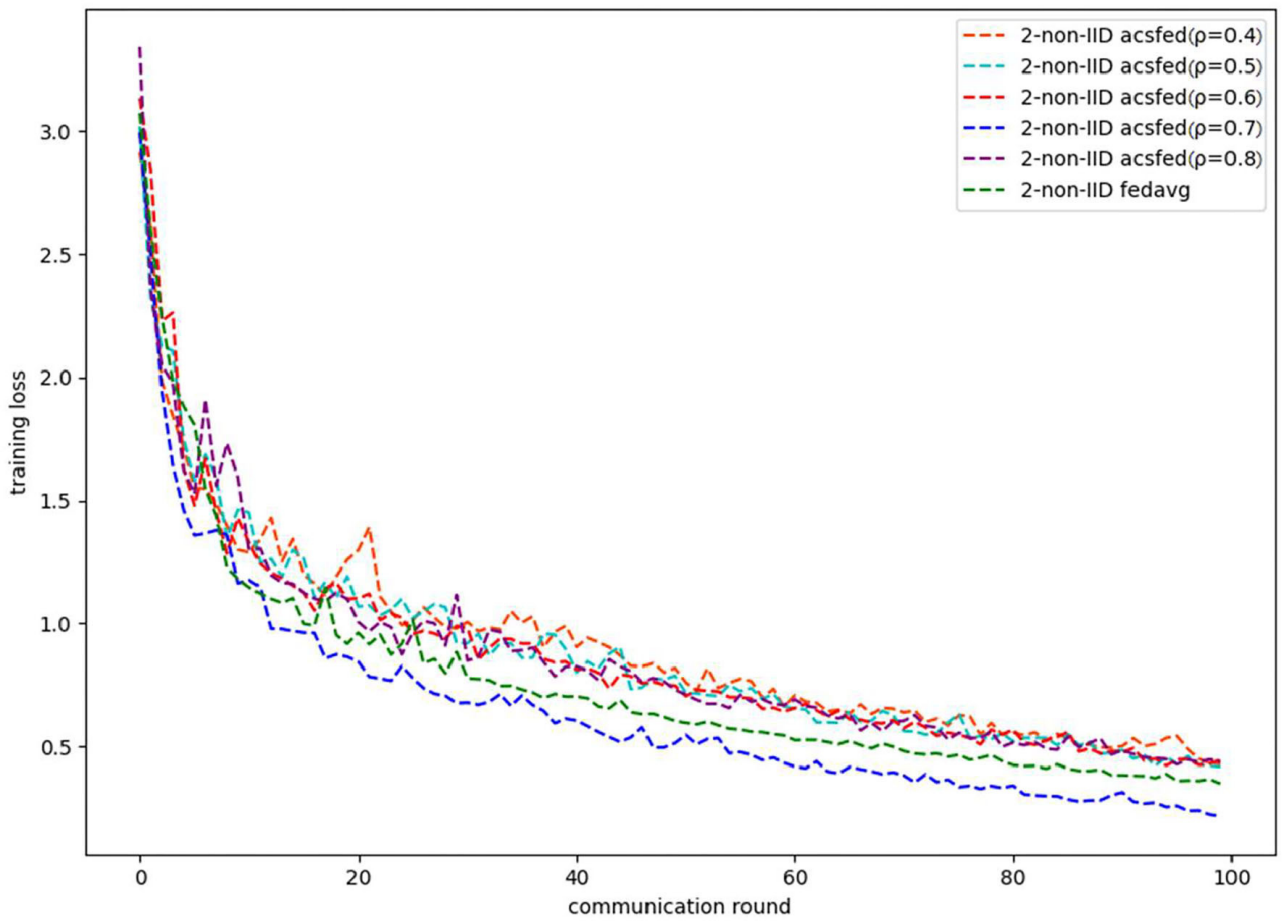
Training loss on MNIST in 2-class non-IID scenario.

**Figure 2:**
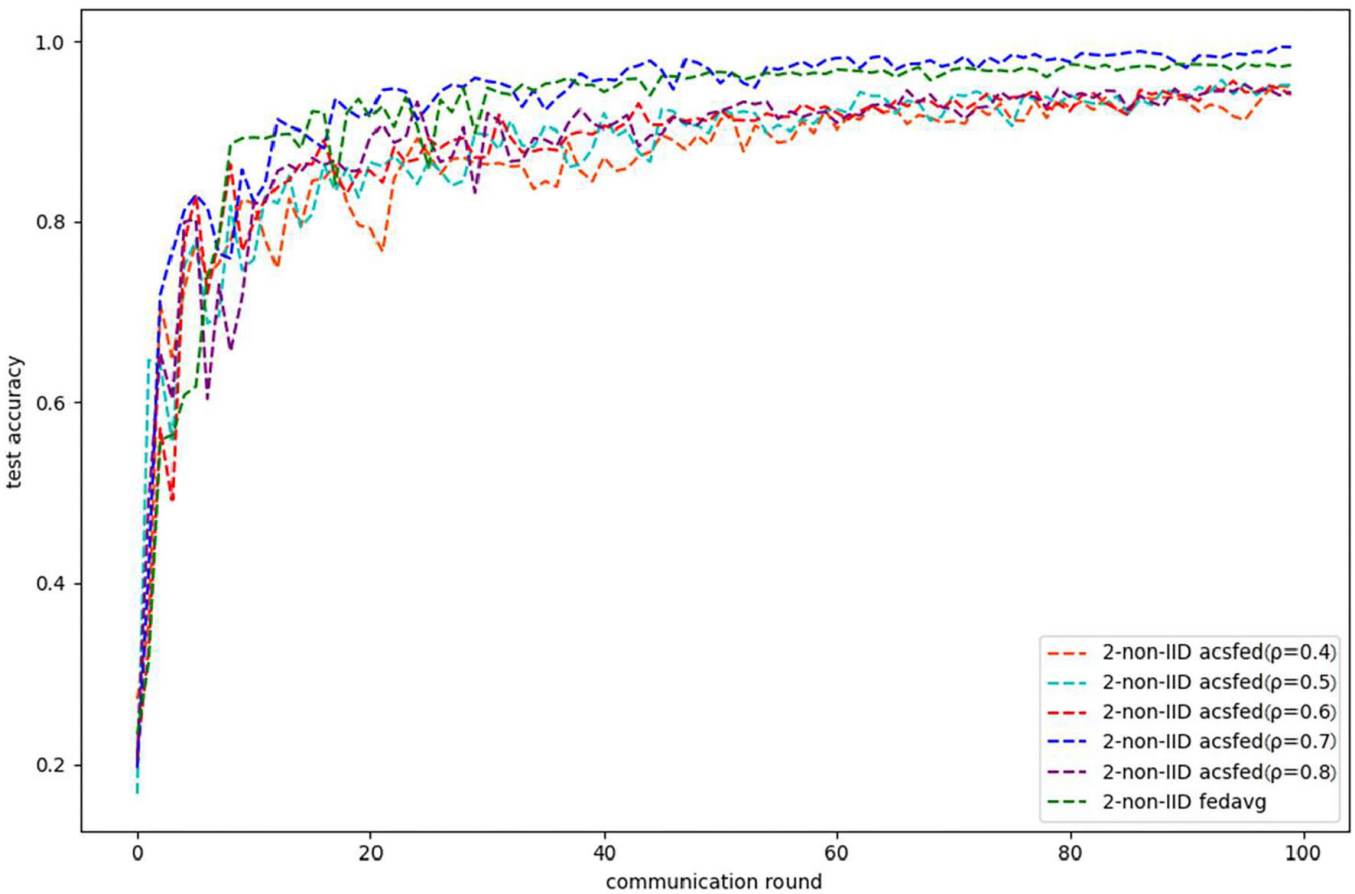
Test accuracy on MNIST in 2-class non-IID scenario.

As shown in Figures 1, 2, ACSFed with attenuation coefficient ρ = 0.7 (blue curve) can achieve lower training loss and higher test accuracy than FedAvg on MNIST in the 2-class non-IID scenario. The trend of the curves in the two figures indicates that the ACSFed training loss decreases faster, and its test accuracy rises faster than FedAvg when ρ = 0.7.

**Figure 3:**
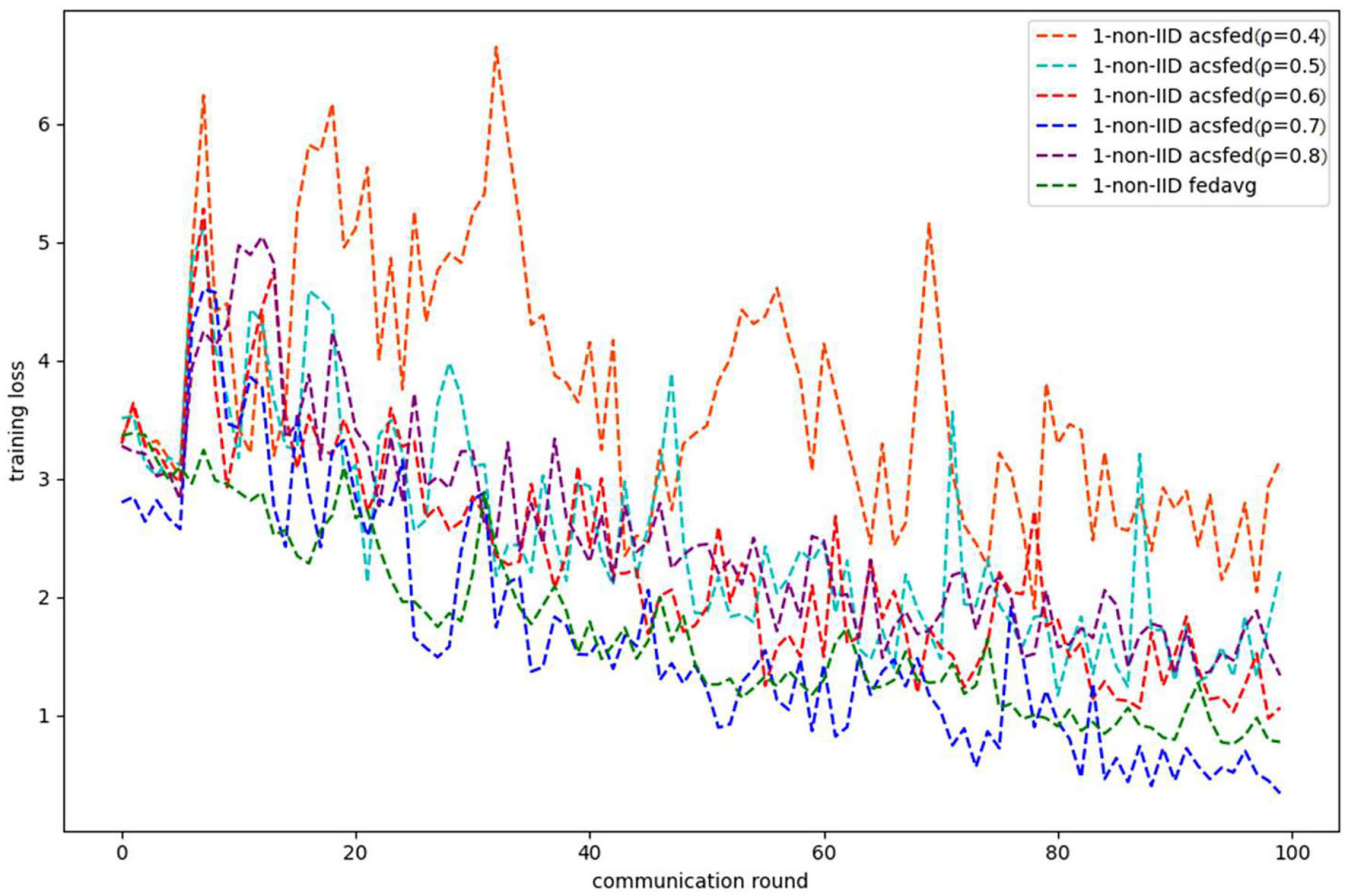
Training loss on MNIST in the 1-class non-IID scenario.

**Figure 4:**
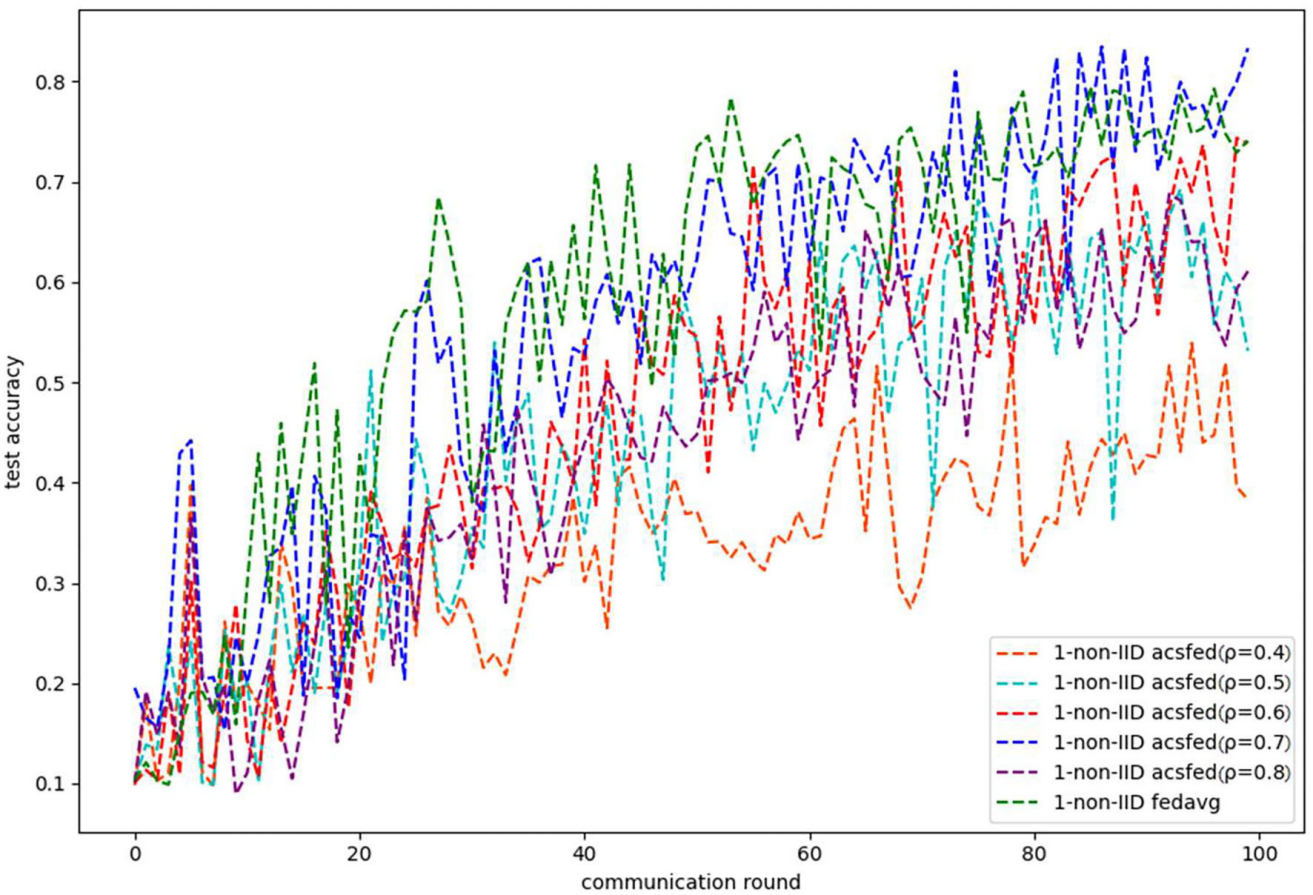
Test accuracy on MNIST in the 1-class non-IID scenario.

Figures 3, 4 show the performance of ACSFed and FedAvg on MNIST in the 1-class non-IID scenario, which has much stronger statistical heterogeneity than the 2-class non-IID scenario; thus, the curves fluctuate markedly. Although the stronger statistical heterogeneity makes it difficult to train the global model, ACSFed ρ = 0.7 can have lower training loss and higher test accuracy than FedAvg.

In addition, the curves of ACSFed will fluctuate much more violently than FedAvg in the earlier communication round due to the client selection strategy of ACSFed that untrained clients and clients with stronger statistical heterogeneity or worse training performance are more likely to be selected, which can feed the model with much more unknown knowledge compared with selecting clients randomly in earlier communication round. However, the ACSFed's loss curve will decrease faster. Its training accuracy curve will increase faster than FedAvg after an earlier communication round as the global model of ACSFed already learned relatively more knowledge than FedAvg in earlier training.

**Figure 5:**
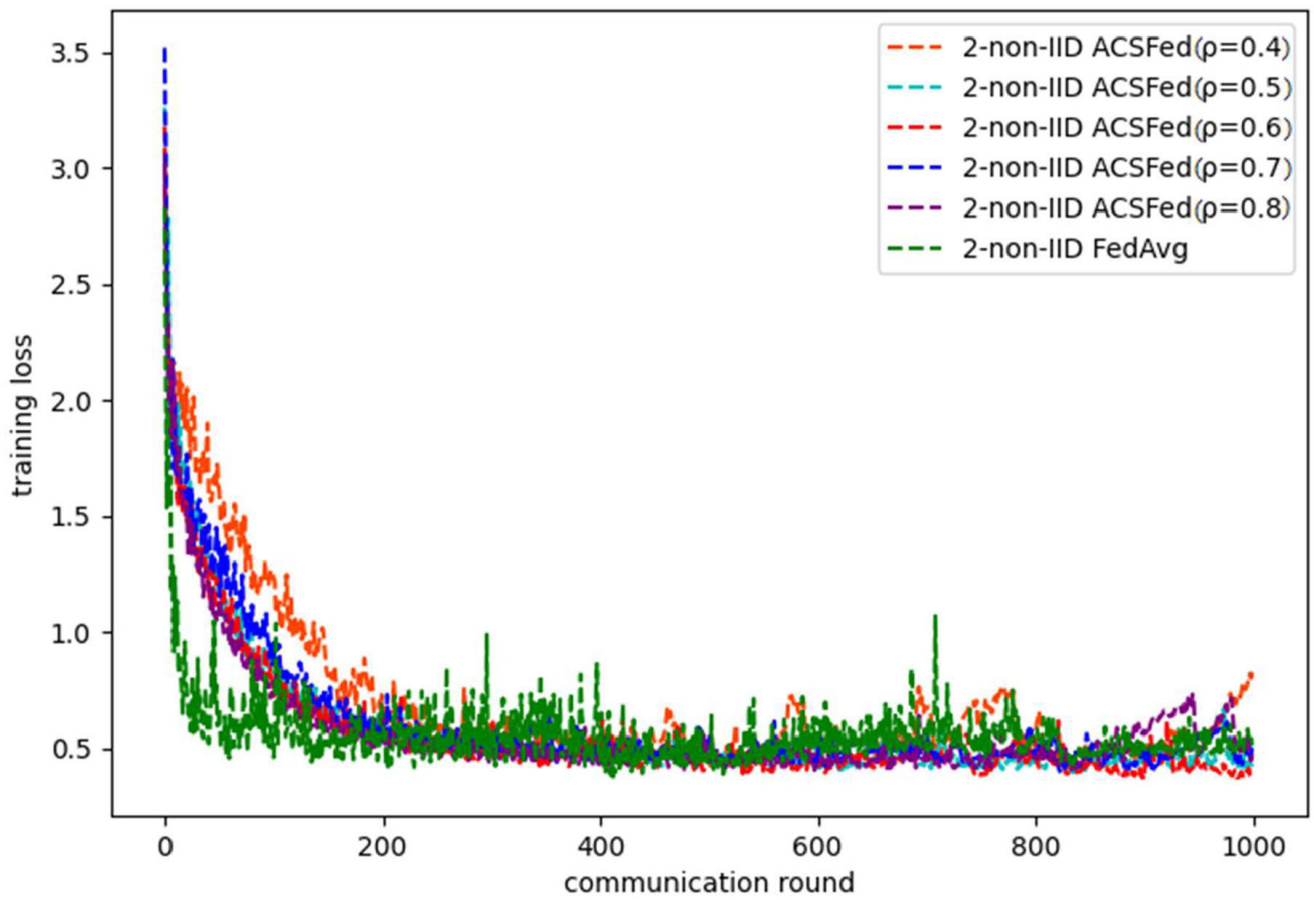
Training loss on fashion MNIST in 2-class non-IID scenario.

**Figure 6:**
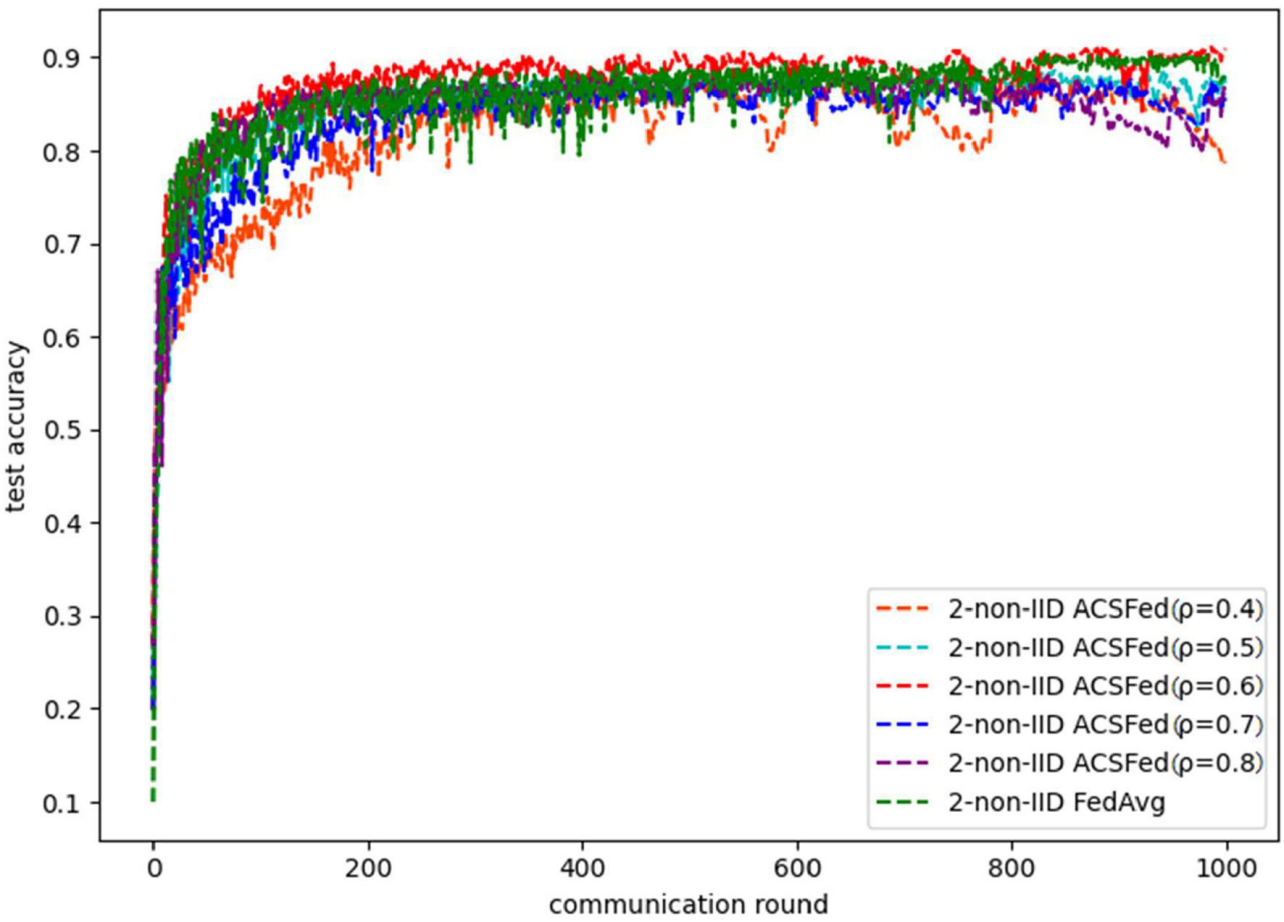
Test accuracy on fashion MNIST in 2-class non-IID scenario.

Similar results in two statistical heterogeneity scenarios can be obtained on Fashion MNIST. In the 2-class non-IID scenario, which is shown by Figures 5, 6, ACSFed with ρ = 0.6 finally achieves a lower training loss and a higher test accuracy than FedAvg. In addition, the client selection strategy will also make the convergence of ACSFed weaker than FedAvg in earlier training rounds. Still, the performance of ACSFed will exceed FedAvg after certain training rounds.

**Figure 7:**
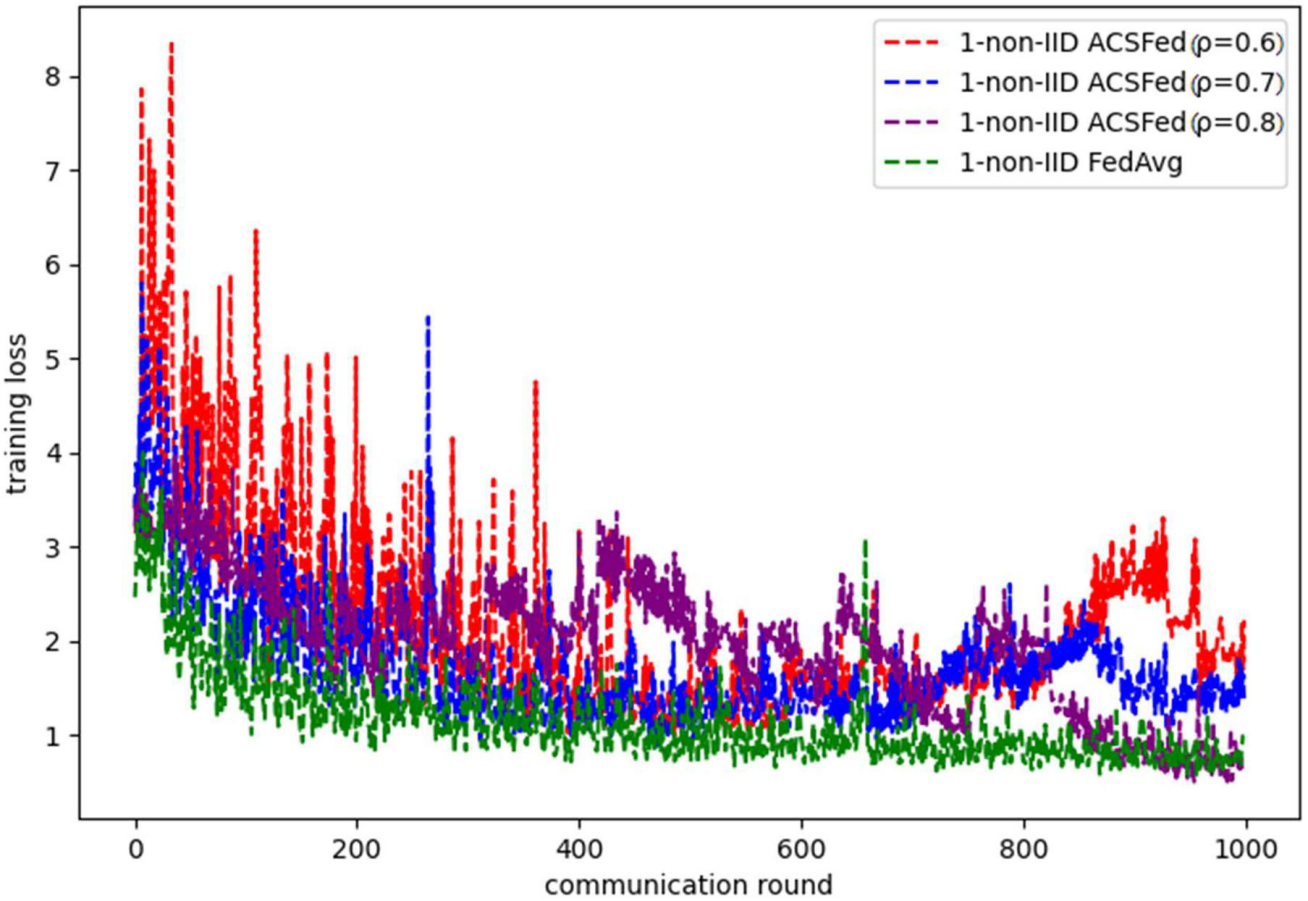
Training loss on fashion MNIST in the 1-class non-IID scenario.

**Figure 8:**
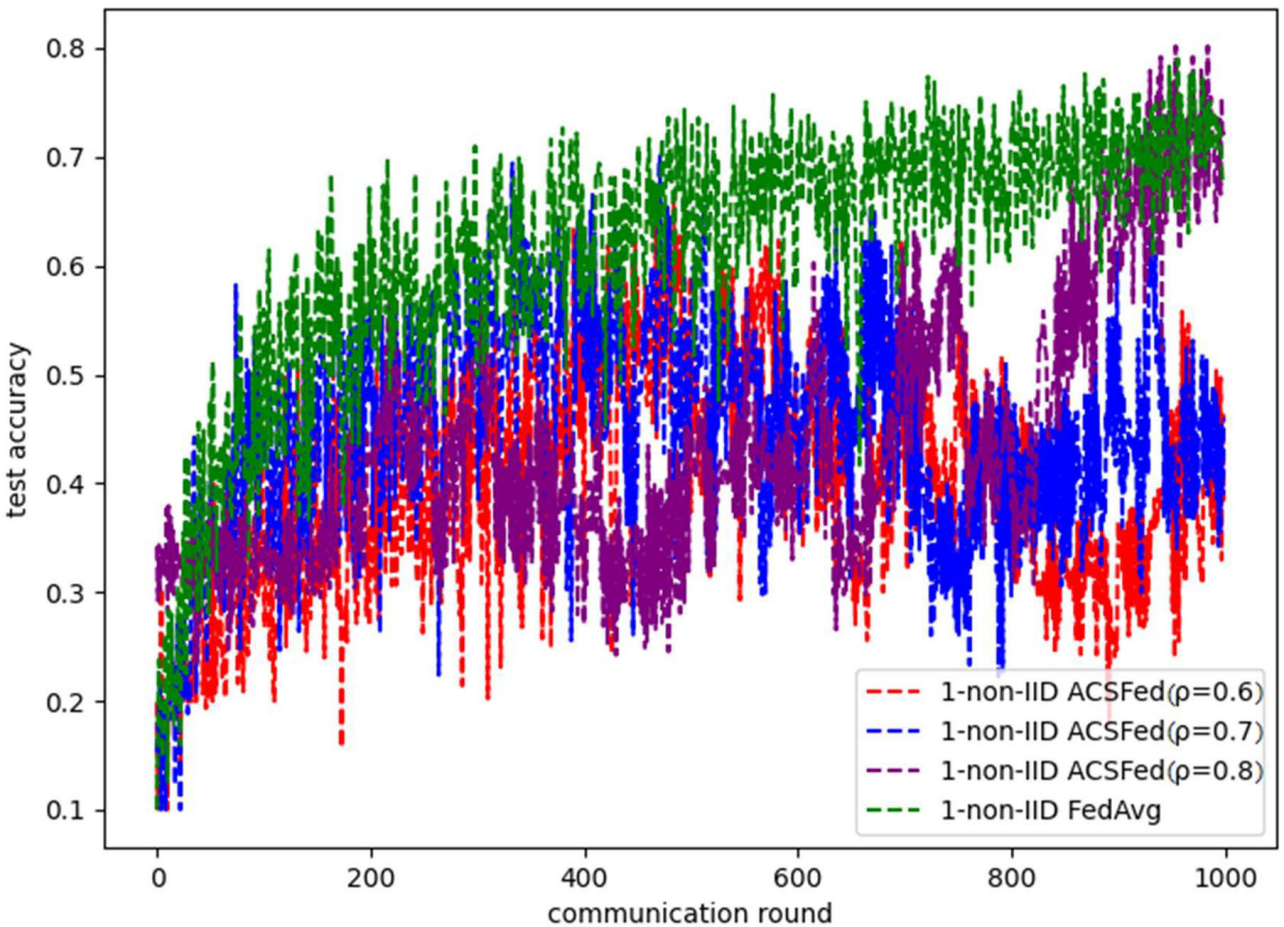
Test accuracy on fashion MNIST in the 1-class non-IID scenario.

However, because Fashion MNIST is more complex than MNIST, ACSFed will take much more time to learn sufficient knowledge about the data in the 1-class non-IID scenario compared to the 2-class non-IID. Results are shown in Figures 7, 8, where ACSFed with ρ = 0.8achieves better performance than FedAvg. In the first 800 rounds of training, the training loss and test accuracy curves of ACSFed fluctuate much more violently than FedAvg. Still, then, a lower loss and higher accuracy than FedAvg is achieved rapidly after 800 rounds of training.

**Figure 9:**
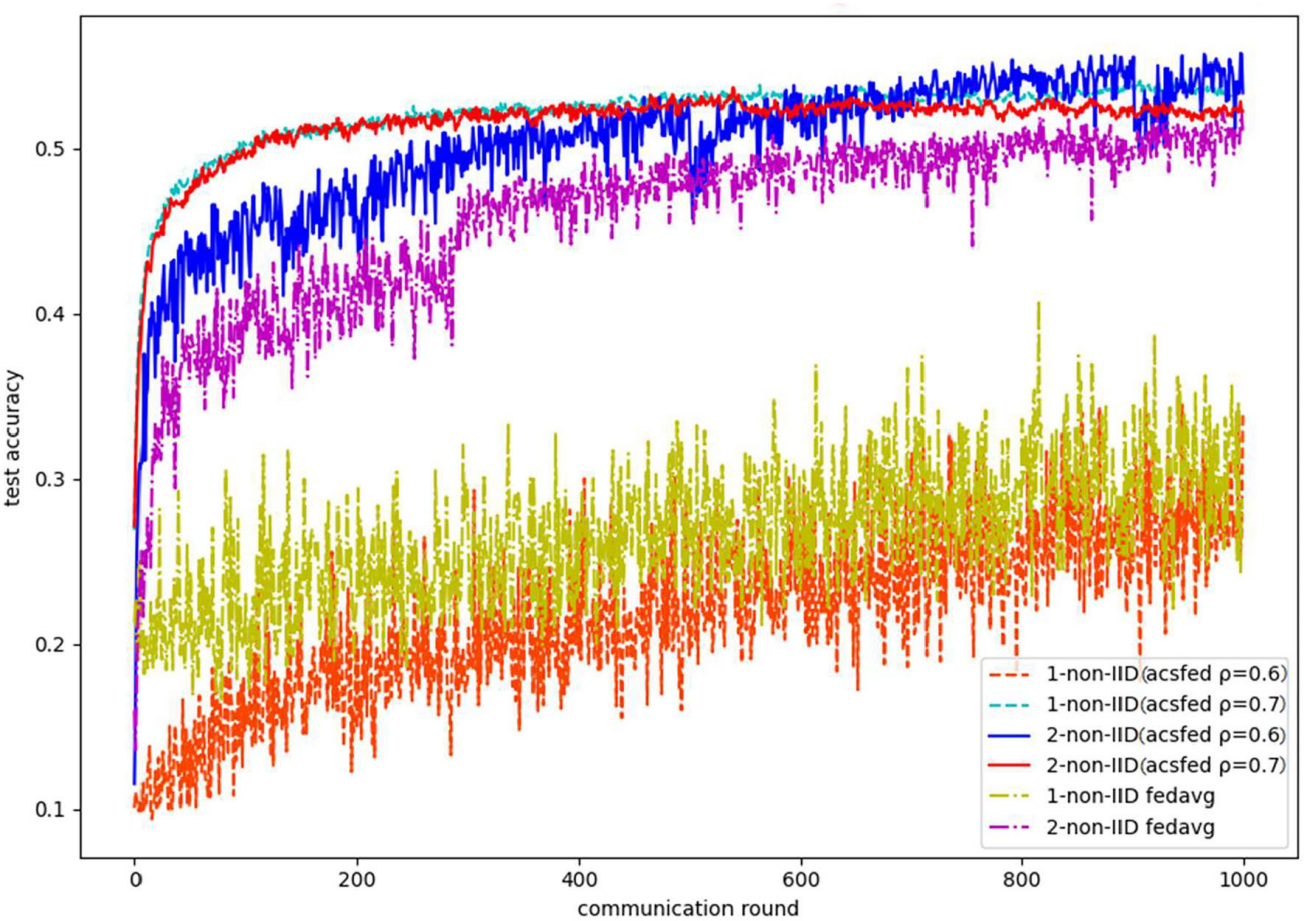
Test accuracy on CIFAR 10 in the 1-class non-IID scenario.

Finally, Figure 9 shows the results on CIFAR10 in 2-class and 1-class non-IID scenarios, which indicate that ACSFed ρ = 0.7 has better performance than FedAvg in two types of statistical scenarios. Moreover, as lite-structured CNN is deployed in the experiment, the accuracy of CIFAR 10 in both scenarios is relatively low. (Complex networks can obtain ideal performance, such as ResNet 18 or ResNet 50, however, these networks are not suitable for a resource-constrained experimental environment.).

**Figure 10:**
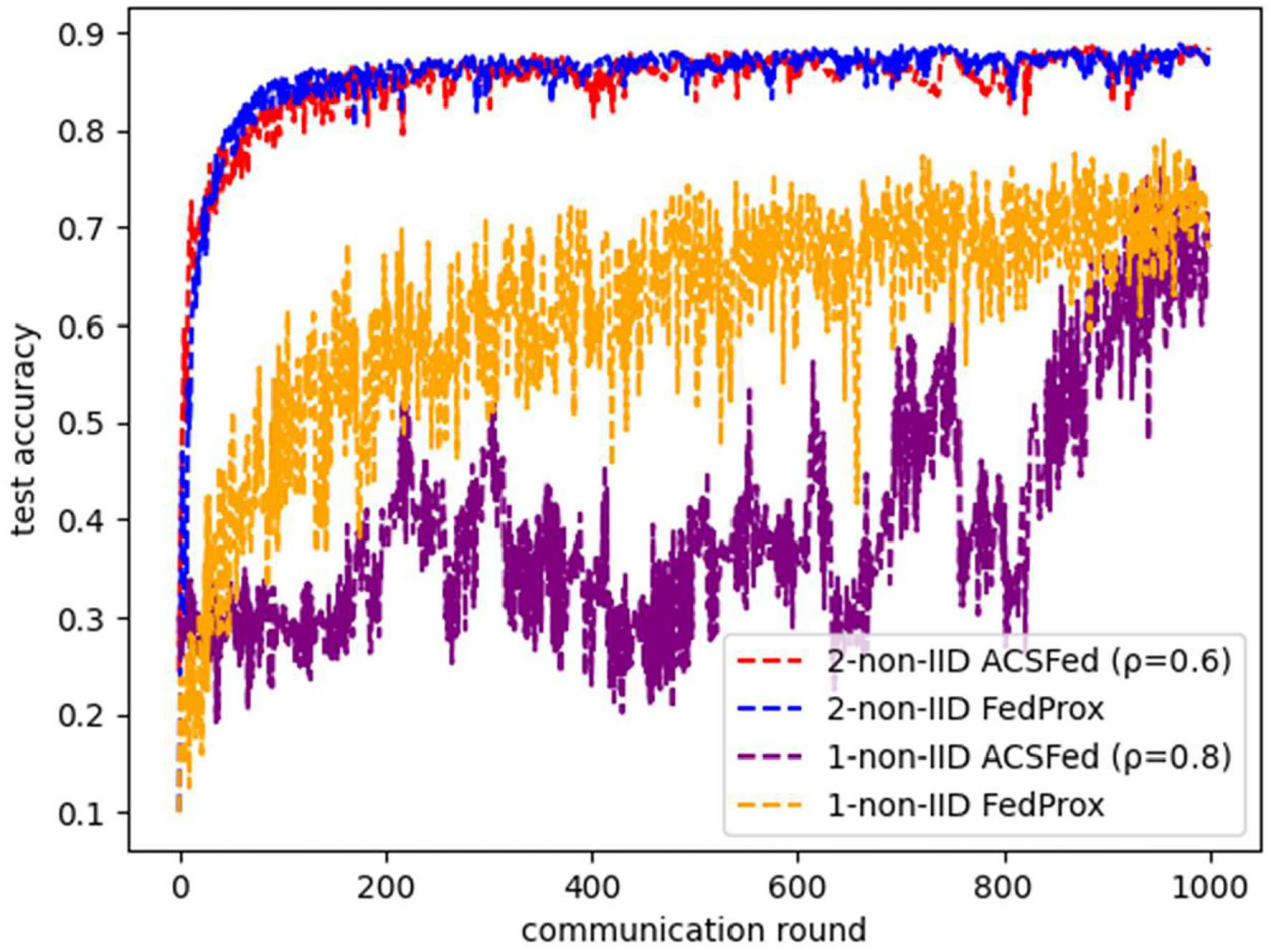
Comparison results between ACSFed and FedProx on fashion MNIST.

Moreover, the comparison result between ACSFed and FedProx is illustrated in Figure 10. It can be concluded that ACSFed can obtain nearly the same promotion in the efficiency of federated learning in both two kinds of statistical heterogeneous scenarios. However, the computational complexity of ACSFed is much smaller than FedProx, as only two types of float digital calculations are added in ACSFed. At the same time, FedProx needs to optimize the divergence between the local model and the global model in the loss function. Therefore, ACSFed can obtain the same performance as FedProx while adding less computational complexity.

Finally, based on these experimental results, the more complex the data are and the stronger statistical heterogeneity is, the more time ACSFed will take to fluctuate in both loss and accuracy; we refer to this time as the fluctuation period, which is due to the client selection strategy of ACSFed. Specifically, the loss and accuracy curve of ACSFed fluctuates more violently than FedAvg in the fluctuation period, indicating that the model keeps learning unknown knowledge. Then, the performance of ACSFed can exceed FedAvg rapidly after the fluctuation period because the model has already learned sufficient knowledge about the non-IID data.

In addition, the ToA@x (the epoch to reach the accuracy of x) of ACSFed and FedAvg are recorded to demonstrate the improvement in training efficiency more clearly, and results are shown in Table 2, where NULL means that the model cannot achieve a certain accuracy after training.

**Table 2:** Training performance of ACSFed and FedAvg.

**Dataset**	**Method**	**ToA@0.6 (2/1 class non-IID)**	**ToA@0.8 (2/1 class non-IID)**
MNIST	ACSFed	3/25	6/72
	FedAvg	5/27	9/NULL
Fashion MNIST	ACSFed	5/108	32/946
	FedAvg	8/107	35/NULL
**–**	**–**	**ToA@0.3** **(2/1 class non-IID)**	**ToA@0.5** **(2/1 class non-IID)**
CIFAR 10	ACSFed	12/381	235/NULL
	FedAvg	20/674	927/NULL

Based on Table 2, ACSFed can achieve a certain accuracy (60 and 80%) more quickly than FedAvg in 1- and 2-class non-IID. In particular, FedAvg cannot achieve an accuracy of 80% in the 1-class non-IID scenario on the three datasets. In comparison, ACSFed can still achieve 80% after a certain communication round of training on MNIST and Fashion MNIST, indicating that the implementation of ACSFed can promote the performance of federated learning in statistical heterogeneity scenarios.

## Conclusion

Federated learning will play an essential role in the future as the computation capability of remote edge devices increases and local data privacy increases. However, statistical heterogeneity can markedly affect the efficiency of federated learning methods and lead to unstable convergence. In this study, we proposed an adaptive client selection algorithm for federated learning called ACSFed to promote the performance of federated learning. Experiments on three different datasets demonstrate the improved performance of ACSFed compared to the current federated learning method. Additionally, ACSFed adds little computation and communication burden because updating the probability matrix of clients is simple. However, there is still the possibility of promoting the performance of federated learning in statistical heterogeneity scenarios by combining ACSFed with methods that focus on global model aggregation. The challenge of model protection still exists, and further research is required to address this and related challenges to apply federated learning methods more effectively.

## Data Availability Statement

Publicly available datasets were analyzed in this study. This data can be found here: http://yann.lecun.com/exdb/mnist/; https://tensorflow.google.cn/datasets/catalog/fashion_mnist; http://www.cs.utoronto.ca/~kriz/cifar.html.

## Author Contributions

AC: conceptualization, methodology, writing—review and editing, and supervision. YF: conceptualization, methodology, data curation, formal analysis, software, and writing—original draft preparation. ZS: conceptualization, methodology, project administration, and investigation. GL: supervision and project administration. All authors contributed to the article and approved the submitted version.

## Funding

This work was supported by National Natural Science Foundation of China (NSFC) (U19A2059), and Sichuan Science and Technology Program (No. 206999977).

## Conflict of Interest

The authors declare that the research was conducted in the absence of any commercial or financial relationships that could be construed as a potential conflict of interest.

## Publisher's Note

All claims expressed in this article are solely those of the authors and do not necessarily represent those of their affiliated organizations, or those of the publisher, the editors and the reviewers. Any product that may be evaluated in this article, or claim that may be made by its manufacturer, is not guaranteed or endorsed by the publisher.

## References

[B1] Asad M. Moustafa A. Ito T. (2020). FedOpt: towards communication efficiency and privacy preservation in federated learning. Appl. Sci. 10, 2864. 10.3390/app10082864

[B2] Cai Z. He Z. (2019). Trading private range counting over big IoT data. In: 2019 IEEE 39th International Conference on Distributed Computing Systems (ICDCS). IEEE, 144–153.

[B3] Cai Z. Zheng X. (2018). A private and efficient mechanism for data uploading in smart cyber-physical systems. IEEE Trans. Network Sci. Eng. 7, 766–775. 10.1109/TNSE.2018.2830307

[B4] Cai Z. Zheng X. Wang J. He Z. (2019). Private data trading towards range counting queries in internet of things. In: IEEE Transactions on Mobile ComputiCng, 2022.

[B5] Chen W. Horvath S. Richtarik P. (2020). Optimal client sampling for federated learning. arXiv [preprint]. arXiv:2010.13723. 10.48550/arXiv.2010.13723

[B6] Cho Y. J. Wang J. Joshi G. (2020). Client selection in federated learning: convergence analysis and power-of-choice selection strategies. arXiv [preprint]. arXiv:2010.0124. 10.48550/arXiv.2010.01243

[B7] Huang L. Yin Y. Fu Z. Zhang S. F. Deng H. Liu D. B. (2020). LoAdaBoost: loss-based AdaBoost federated machine learning with reduced computational complexity on IID and non-IID intensive care data. PLoS ONE 15, e0230706. 10.1371/journal.pone.0230706 32302316 PMC7164603

[B8] Jeong E. Oh S. Kim H. Park J. Bennis M. Kim S. (2018). Communication-efficient on-device machine learning: Federated distillation and augmentation under non-iid private data. arXiv [preprint]. arXiv:1811.11479. 10.48550/arXiv.1811.11479

[B9] Konečný J. McMahan B. Ramage D. (2015). Federated optimization: distributed optimization beyond the datacenter. arXiv [preprint]. arXiv:1511.03575. 10.48550/arXiv.1511.03575

[B10] Konečný J. McMahan H. B. Yu F. X. Richtárik P. Bacon D. (2016). Federated learning: strategies for improving communication efficiency. arXiv [preprint]. arXiv:1610.05492. 10.48550/arXiv.1610.05492

[B11] Li D. Wang J. (2019). Fedmd: Heterogenous federated learning via model distillation. arXiv [preprint]. arXiv:1910.03581. 10.48550/arXiv.1910.03581

[B12] Li T. Sahu A. K. Zaheer M. Sanjabi M. Talwalkar A. Smith V. (2020). “Federated optimization in heterogeneous networks,” in Proceedings of Machine Learning and Systems (Austin, TX), 429–450.

[B13] Li X. Huang K. Yang W. Wang S. S. Zhang Z. H. (2019). On the convergence of fedavg on non-iid data. arXiv [preprint]. arXiv:1907.02189. 10.48550/arXiv.1907.0218933296314

[B14] McMahan B. Moore E. Ramage D. Hampson S. Arcas B. A. Y. (2017a). “Communication-efficient learning of deep networks from decentralized data,” in Proceedings of the 20th International Conference on Artificial Intelligence and Statistics (Ft. Lauderdale, FL), 1273–1282.

[B15] McMahan B. Ramage D. Research Scientists. (2017b). Federated learning: Collaborative machine learning without centralized training data. Google AI Blog. Available online at: https://ai.googleblog.com/2017/04/federated-learning-collaborative.html

[B16] Nishio T. Shinkuma R. Takahashi T. Mandayam N. (2013). Service-oriented heterogeneous resource sharing for optimizing service latency in mobile cloud. In: Proceedings of the First International Workshop on Mobile Cloud Computing & Networking, 19–26.

[B17] Nishio T. Yonetani R. (2019). lient selection for federated learning with heterogeneous resources in mobile edge. In: ICC 2019-2019 IEEE International Conference on Communications (ICC). IEEE, 1–7.

[B18] Pang J. Huang Y. Xie Z. Hang Q. L. Cai Z. P. (2020). Realizing the heterogeneity: a self-organized federated learning framework for IoT. IEEE Internet Things J. 8, 3088–3098. 10.1109/JIOT.2020.3007662

[B19] Sardellitti S. Scutari G. Barbarossa S. (2015). Joint optimization of radio and computational resources for multicell mobile-edge computing. IEEE Trans. Signal Inform. Process. Over Netw. 1, 89–103. 10.1109/TSIPN.2015.2448520

[B20] Sattler F. Wiedemann S. Müller K. R. Samek W. (2019). Robust and communication-efficient federated learning from non-iid data. IEEE Trans. Neural Networks Learn. Syst. 31, 3400–3413. 10.1109/TNNLS.2019.2944481 31689214

[B21] Shen G. Gao D. Yang L. Zhou F. Song D. X. Lou W. . (2022). Variance-reduced heterogeneous federated learning via stratified client selection. arXiv [preprint]. arXiv:2201.05762. 10.48550/arXiv.2201.05762

[B22] Tieleman T. Hinton G. (2012). Lecture 6.5-rmsprop: Divide the gradient by a running average of its recent magnitude. COURSERA. 4, 26–31.

[B23] Wang H. Kaplan Z. Niu D. Li B. C. (2020). Optimizing federated learning on non-iid data with reinforcement learning. In: IEEE INFOCOM 2020-IEEE Conference on Computer Communications. IEEE, 1698–1707.

[B24] Wang S. Tuor T. Salonidis T. Leung K. Makaya C. He T. . (2019). Adaptive federated learning in resource constrained edge computing systems. IEEE J. Select. Areas Commun. 37, 1205–1221. 10.1109/JSAC.2019.2904348

[B25] Xiong Z. Cai Z. Takabi D. Li W. (2021). Privacy threat and defense for federated learning with non-iid data in AIoT. IEEE Trans. Indus. Inform. 18, 1310–1321. 10.1109/TII.2021.3073925

[B26] Yu Y. Zhang J. Letaief K. B. (2016). Joint subcarrier and CPU time allocation for mobile edge computing. In: 2016 IEEE Global Communications Conference (GLOBECOM). IEEE, 1–6.

[B27] Zhang M. Sapra K. Fidler S. Yeung S. Alvarez J. M. (2020). Personalized federated learning with first order model optimization. arXiv [preprint]. arXiv:2012.08565. 10.48550/arXiv.2012.08565

[B28] Zhang S. Q. Lin J. Zhang Q. (2022). A multi-agent reinforcement learning approach for efficient client selection in federated learning. arXiv [preprint]. arXiv:2201.02932. 10.48550/arXiv.2201.02932

[B29] Zhang W. Wang X. Zhou P. Wu W. Zhang X. (2021). Client selection for federated learning with non-iid data in mobile edge computing. IEEE Access 9, 24462–24474. 10.1109/ACCESS.2021.3056919

[B30] Zhao J. Feng Y. Chang X. Chang X. Liu C. (2022). Energy-efficient client selection in federated learning with heterogeneous data on edge. Peer Peer Netw. Appl. 15, 1–13. 10.1007/s12083-021-01254-8

[B31] Zhao Y. Li M. Lai L. Suda N. Civin D. Chandra V. (2018). Federated learning with non-iid data. arXiv [preprint]. arXiv:1806.00582. 10.48550/arXiv.1806.00582

